# Supplementary material for: Phosphorylation of Xenopus M18BP1 governs centromeric localization and CENP-A nucleosome assembly
Source: EMBO Rep. 2026 Feb 12;27(6):1561–79. doi: 10.1038/s44319-026-00714-7 (PMC13021935; doi:10.1038/s44319-026-00714-7)
Supplement: Supplementary file 2 — Expanded View Figures [file 44319_2026_714_MOESM2_ESM.pdf]

Expanded View Figures

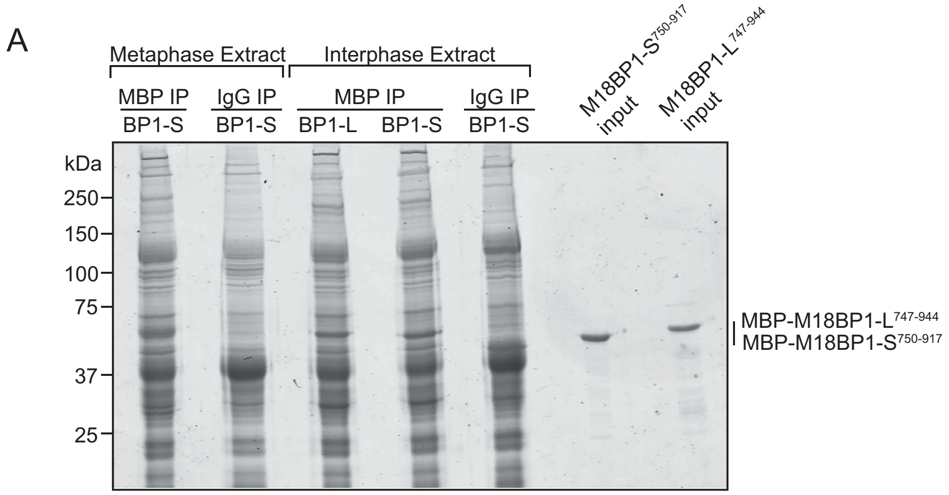

B

Metaphase

M18BP1-L amino acid	% modified	Cdk?
T814	100.00%	Y
S936	100.00%	Y
S876	78.98%	Y
S749	64.50%	Y
S760	63.79%	N
S822	57.57%	N
T882	49.84%	Y
S838	43.74%	N
S888	40.63%	N

Interphase

M18BP1-L amino acid	% modified	Cdk?
S876	18.75%	Y
T861	5.77%	N
S864	2.34%	N
T866	2.24%	N
S828	0.22%	N
S760	0.00%	N

C

Metaphase

M18BP1-S amino acid	% modified	Cdk?
S841	95.08%	N
T826	48.43%	Y
S825	39.02%	N
S873	15.24%	N
S834	2.50%	Y
S869	2.32%	N
S889	1.68%	N

Interphase

M18BP1-S amino acid	% modified	Cdk?
S878	0.12%	N
S889	0.06%	N

Figure EV1. Mass spectrometry of M18BP1 from interphase or metaphase egg extract.

(**A**) SDS-PAGE gel of MBP-M18BP1-S$^{750\text{-}917}$ and MBP-M18BP1-L$^{747\text{-}944}$ samples submitted for mass spectrometry. Metaphase egg extract was supplemented with *X. laevis* MBP-M18BP1-S$^{750\text{-}917}$ and interphase egg extract was supplemented with *X. laevis* MBP-M18BP1-S$^{750\text{-}917}$ and MBP-M18BP1-L$^{747\text{-}944}$, then immunoprecipitated and submitted for mass spectrometry. Mock IgG immunoprecipitation samples are shown, as well as input MBP-M18BP1-S$^{750\text{-}917}$ and MBP-M18BP1-L$^{747\text{-}944}$ protein. (**B**) Metaphase table adapted from French et al, 2017, displaying the nine most abundant phosphorylation events of M18BP1-L in metaphase egg extract detected with mass spectrometry. Interphase table displays data collected in this manuscript, for all phosphorylation events of M18BP1-L in interphase egg extract detected with mass spectrometry. Estimated abundance of each residue is shown, and green highlighting indicates presence of a Cdk consensus motif (S/T-P). (**C**) Tables displaying the phosphorylation events of M18BP1-S in metaphase or interphase egg extract detected with mass spectrometry. Estimated abundance of each residue is shown, and green highlighting indicates presence of a Cdk consensus motif (S/T-P).

© The Author(s)

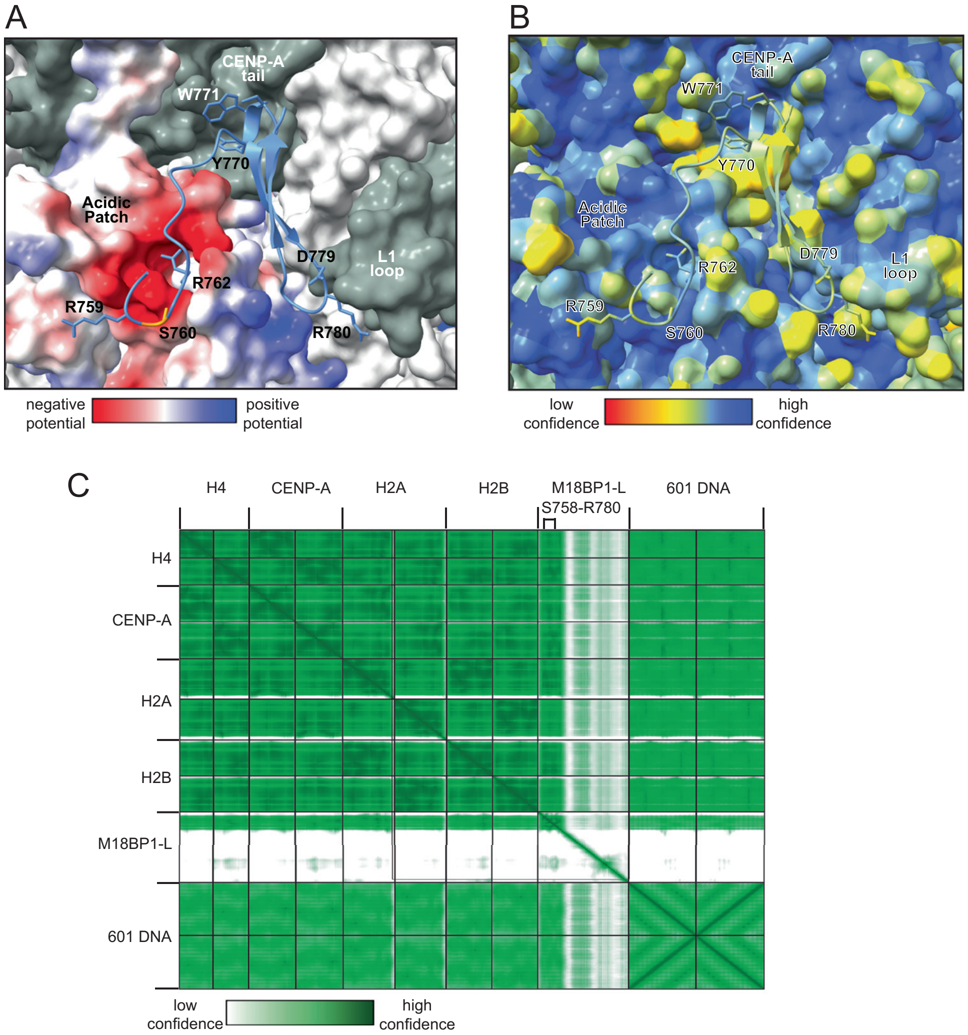

Figure EV2. AlphaFold model of M18BP1-L bound to CENP-A.

(**A**) AlphaFold structural model of the *X. laevis* CENP-A nucleosome bound to M18BP1-L$^{758-789}$. The surface model of the C-term tail and L1 loop of the CENP-A nucleosome is shown in dark gray and the surface model of the acidic patch of H2A/H2B is colored by electrostatic potential, with red depicting negative potential and blue depicting positive potential. M18BP1-L is shown in blue, with the conserved residues shown to interact with the CENP-A nucleosome in *G. gallus* labeled (Jiang et al, 2023) and residue S760 highlighted in orange. (**B**) The same AlphaFold structural model as Fig. EV3A, however the model is colored according to the pLDDT local confidence value (low confidence is red, and high confidence is blue). (**C**) Predicted Aligned Error (PAE) plot depicting global confidence of the AlphaFold structural model depicted in Fig. 1B and Fig. EV2A,B. Location of the individual proteins and 601 DNA sequence are depicted along the top and left-hand side of the plot. The residues S758-R780 of M18BP1-L are labeled along the top of the plot.

 © The Author(s)

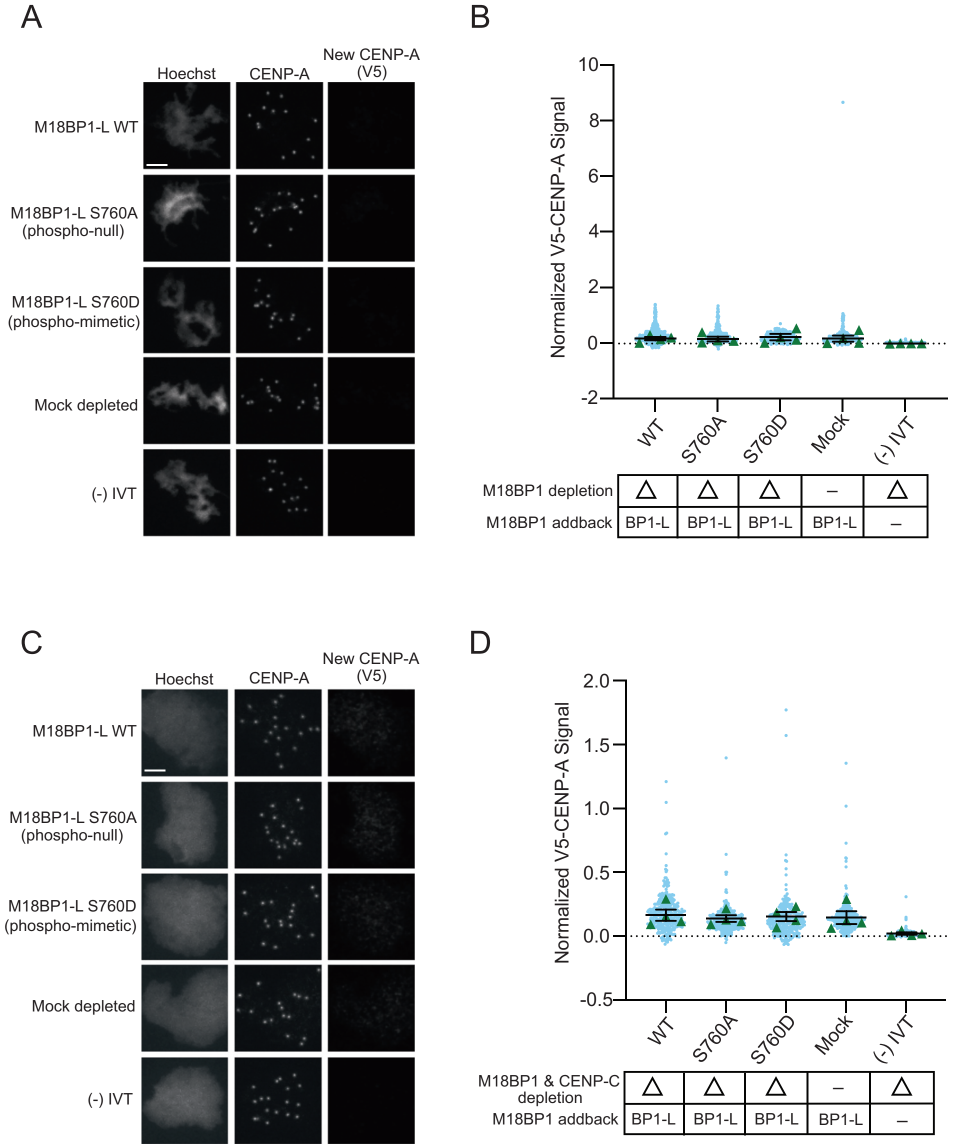

Figure EV3. M18BP1-L phospho-mutants do not support metaphase CENP-A assembly.

(**A**) Representative immunofluorescence images of new V5-CENP-A assembly in metaphase extract immunodepleted of endogenous M18BP1 then supplemented with full-length WT or mutant FLAG-M18BP1-L or a mock depletion or (−) IVT control (indicated on left). Labeling for DNA (Hoechst), total CENP-A, and new CENP-A (V5) is indicated above the image. Scale bar is 5 μm. (**B**) Quantification of new V5-CENP-A with controls (indicated below) in metaphase egg extract immunodepleted of endogenous M18BP1. M18BP1 depletion and addback condition is indicated in the bottom table. The signal is normalized to the WT FLAG-M18BP1-L addback condition. Error bars represent SEM of four independent replicates ($n = 4$) with green triangles displaying the mean of each replicate and blue circles representing each individual centromere. (**C**) Representative immunofluorescence images of new V5-CENP-A assembly in metaphase extract immunodepleted of endogenous CENP-C and M18BP1 then supplemented with full-length WT or mutant FLAG-M18BP1-L or a mock depletion or (−) IVT control (indicated on left). Labeling for DNA (Hoechst), total CENP-A, and new CENP-A (V5) is indicated above the image. Scale bar is 5 μm. (**D**) Quantification of new V5-CENP-A with controls (indicated below) in metaphase egg extract immunodepleted of endogenous CENP-C and M18BP1. CENP-C and M18BP1 depletion and addback condition is indicated in the bottom table. The signal is normalized to the WT FLAG-M18BP1-L addback condition. Error bars represent SEM of four independent replicates ($n = 4$) with green triangles displaying the mean of each replicate and blue circles representing each individual centromere.
